# Enhancing the Antibacterial and Biointegrative Properties of Microporous Titanium Surfaces Using Various Metal Coatings: A Comparative Study

**DOI:** 10.3390/prosthesis7060133

**Published:** 2025-10-26

**Authors:** Maxim Shevtsov, Ekaterina Bozhokina, Natalia Yudintceva, Danila Bobkov, Anastasiya Lukacheva, Denis Nazarov, Irina Voronkina, Larisa Smagina, Emil Pitkin, Elena Oganesyan, Airat Kayumov, Grigory Raykhtsaum, Mykhailo Matviychuk, Vladimir Moxson, Michael Akkaoui, Stephanie E. Combs, Mark Pitkin

**Affiliations:** 1 Klinikum rechts der Isar, Technical University of Munich, 81675 Munich, Germany; 2 Institute of Cytology of the Russian Academy of Sciences (RAS), 194064 Saint Petersburg, Russia; 3 Saint Petersburg State Pediatric Medical University, Litovskaya Str. 2.,194100 Saint Petersburg, Russia; 4 Saint Petersburg State University, Universitetskaya Nab, 7/9, 199034 Saint Petersburg, Russia; 5 Institute of Experimental Medicine, Acad. Pavlov Str. 12., 197022 Saint Petersburg, Russia; 6 Department of Statistics and Data Science, The Wharton School, University of Pennsylvania, Philadelphia, PA 19104, USA; 7 Institute of Fundamental Biology and Medicine, Kazan Federal University, 420012 Kazan, Russia; 8 Poly-Orth International, Sharon, MA 02067, USA; 9 ADMA Products, Inc., Hudson, OH 44236, USA; 10 Tanury Industries, Lincoln, RI 02865, USA; 11 Department of Orthopaedics and Rehabilitation Medicine, Tufts University School of Medicine, Boston, MA 02111, USA

**Keywords:** direct skeletal attachment, skin- and bone-integrated pylon, SBIP, metal coatings, silver (Ag) coating, titanium nitride (TiN) coating, zirconium nitride (ZrN) coating, copper (Cu) coating, titanium implants

## Abstract

**Background/Objectives:**

A comparative study of silver (Ag), titanium nitride (TiN), zirconium nitride (ZrN), and copper (Cu) coatings on titanium (Ti) disks, considering the specifications of a microporous skin- and bone-integrated titanium pylon (SBIP), was performed to assess their biocompatibility, osseointegration, and mechanical properties.

**Methods:**

To assess cytotoxicity and biocompatibility, Ti disks with various metal coatings were co-cultured with FetMSCs and MG-63 cells for 1, 3, 7, and 14 days and subsequently evaluated using a cell viability assay, as supported by SEM and confocal microscopy studies. The antimicrobial activity of the selected four materials coating the implants was tested against *S. aureus* by mounting Ti disks onto the surface of LB agar dishes spread with a bacterial suspension and measuring the diameter of the growth inhibition zones. Quantitative Real-Time Polymerase Chain Reaction (RT-PCR) analysis of the relative gene expression of biomarkers that are associated with extracellular matrix components (fibronectin, vitronectin, type I collagen) and cell adhesion (α2, α5, αV integrins), as well as of osteogenic markers (osteopontin, osteonectin, TGF-β1, SMAD), was performed during the 14-day follow-up period. Additionally, the activity of matrix metalloproteinases (MMP-1, -2, -8, -9) was assessed.

**Results:**

All samples with metal coatings, except the copper coating, demonstrated a good cytotoxicity profile, as evidenced by the presence of a cellular monolayer on the sample surface on the 14th day of the follow-up period (as shown by SEM and inverted confocal microscopy). All metal coatings enhanced MMP activity, as well as cellular adhesion and osteogenic marker expression; however, TiN showed the highest values of these parameters. Significant inhibition of bacterial growth was observed only in the Ag-coated Ti disks, and it persisted for over 35 days.

**Conclusions:**

The silver-based coating, due to its high antibacterial activity, low cytotoxicity, and biointegrative capacity, can be recommended as the coating of choice for microporous titanium implants for further preclinical studies.

## 1. Introduction

The technology of direct skeletal attachment (DSA) for exoprosthetics of limbs has already entered the practice of traumatological and orthopedic clinics, having proven itself as a reliable approach for the rehabilitation of patients with a high level of preservation of limb biomechanics (as compared with conventional socket prostheses) and, consequently, improved functional integration and quality of life [1]. A pylon is implanted into the bone’s intramedullary canal and exits through the skin, to which the exoprosthetic structure is then attached. Reliable bone fixation and biointegration of the intraosseous pylon can be achieved, as shown by numerous preclinical and clinical studies [2,3]. At the same time, the presence of a percutaneous section of the implant passing through the soft tissues of the residuum can be considered as an entry point for infection, resulting in different postoperative complications [4,5]. One approach to mitigating infectious complications is the development of an implant that allows cells to grow into its walls. This approach has been intensively investigated by the authors’ team and implemented in the design of the skin- and bone-integrated pylon (SBIP) with micropores, allowing bone tissue and skin cells to effectively migrate into the pores of the implant, forming a vascularized strong tissue barrier against infection [6]. Thus, in our recent study, it was demonstrated that micropore sizes ranging from 200 to 500 μm in titanium 3D-printed implants were favorable for dermal fibroblast adhesion, while pore sizes ranging from 400 to 800 μm demonstrated favorable results (in terms of biomarker expression related to osteogenic differentiation, including osteonectin, osteocalcin, osteopontin, SMAD4, and TGF-β1) in osteoblast cells [7]. These parameters create favorable conditions for reliable mechanical fixation of the implant and help close entry sites for infection.

Another approach for mitigating infection complications is the creation of an optional antibacterial coating that prevents the formation of a biofilm and colonization of the implant surface by bacteria [8,9].

It is this approach to which the current study is devoted. We conducted a comparative analysis of the use of antibacterial coatings on implants based on various metals (silver (Ag), titanium nitride (TiN), zirconium nitride (ZrN), and copper (Cu), which have proven to be cost-effective and reliable in traumatology and orthopedics. Materials other than silver that are potentially useful for accelerated cell growth and have been tested in various studies [10,11] include titanium nitride (TiN) and zirconium nitride (ZrN) [12–14], as well as copper (Cu) [15].

Considering that the use of metal-based coatings can significantly affect the biointegrative properties of porous titanium materials, namely, the ingrowth of bone and skin tissue cells into the pores of the implant, we investigated in vitro the effect of these coatings on both the viability and functional properties of model cells—FetMSCs and MG-63 cells. To study the properties of the cells, we selected cell adhesion markers (α2 integrin (collagen-specific), α5 integrin (fibronectin-specific), αV integrin (vitronectin-specific), type I collagen, fibronectin, vitronectin, FAK, vinculin, paxillin), osteogenic markers (osteonectin, osteopontin, TGF-β1, SMAD), and the activity of matrix metalloproteinases (i.e., activity of gelatinases (MMP-2, MMP-9) and collagenases (MMP-1, MMP-8)).

According to the results of the study, it was found that coatings based on copper and silver have the best antibacterial properties; however, due to the cytotoxic activity of copper and the effect on the functional properties of cells, the silver coating is favorable.

## 2. Materials and Methods

### 2.1. Synthesis, Metal Coating, and Characterization of the Ti Disks

#### 2.1.1. Porous Titanium Samples for the Current Study

**Figure 1.**
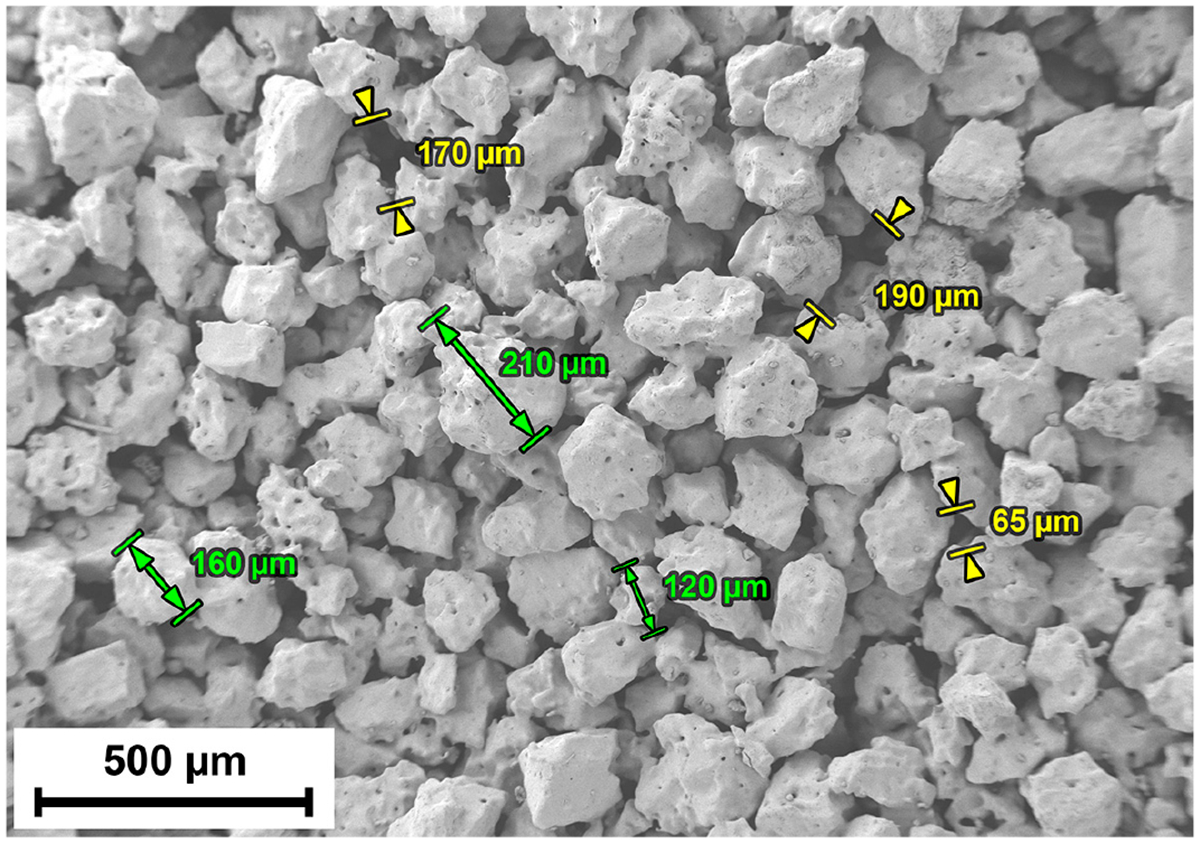
Structure of the sintered (control) samples.

Porous titanium tablet samples (Figure 1) were fabricated with sintering technology (ADMA Products Group, Hudson, OH, USA) from surgical implant-grade titanium (ASTM F67) with pore sizes between 20 μm and 350 μm [16], in line with Poly-Orth International Standard Operating Procedure MPPS-103. Sintering was conducted using molds made of boron nitride block with cylindrical cavities with a diameter of 8.5 mm and a depth of 3 mm. The cavities were filled with high-purity titanium hydride powder that was pre-sieved to *−*50/+80 mesh (ASTM E11).

The sintering process consisted of two stages as follows:

1. Once the 10^*−*5^ torr vacuum was achieved, the chamber was back-filled with high-purity argon to partial pressure between 11 psi and 12 psi. The sintering (that involved decomposition of titanium hydride to pure titanium) was carried out at 1190 °C (2174 °F) for 2 h.
2. For the second stage of sintering, the vacuum pressure was reduced to 10^*−*5^ torr (no argon), and the temperature was raised to 1300 °C (2372 °F). The sintering time was 4 h.

The tablets were removed from the cavities and then subjected to hot isostatic pressing with argon pressure of 15,000 psi for 2 h at a temperature of 954 °C (1749 °F).

We used a patented combination of four key technological characteristics: porosity, pore size, porosity volume fraction, and particle size [16]. The parameter that is most distinct from other implants’ systems is the porosity volume fraction (*VF*), which is the fraction of void space relative to the total bulk volume of the sample. In our samples, *VF* = 78.2%. This value is associated with implants with deep porosity (*VF* > 50%), as defined in [6].

#### 2.1.2. Coating of the Samples (Figure 2)

The properties of the materials that are potentially useful for the accelerated cell growth are as follows: (a) natural antibacterial properties, (b) biocompatibility, and (c) if prepared properly either by composition or multilayered composites, the antibacterial components shedding quickly to prevent infection before cell growth initiates. In addition to silver, TiN, ZrN, and Cu have been trialed in various studies [13,17–20]. Each of these materials has its potential benefits and drawbacks.

Copper composites require a limited amount of copper, as copper ions can be antibacterial, but where too much copper metal is exposed to the body, it becomes an irritant causing infection [21]. For Cu, the composite aspect of the application is critical, but the technology required is in the laboratory phase, at best. While TiN and ZrN are excellent materials, they do not possess the full antibacterial properties of silver [12–14].

The technology used for coating was physical vapor deposition (PVD) [22], and the equipment used was a magnetron sputtering multi-target machine, Flexicoat series (IHI Hauzer Techno Coating B.V., Venlo, The Netherlands).

For all metals used for coating, the tablets were thoroughly cleaned with aqueous-base soap and ultrasonic equipment. Once the ultrasonic process was completed, the soap film on the tablets was removed and the tablets neutralized using a mild sulfuric acid, followed by rinsing in DI water. The tablets were then dried at 300 F for about 60 min to ensure all the water vapor was completely removed from the tablet. This method ensured two critical elements in the physical vapor deposition (PVD) process: (1) there were no organics on the tablet to cause poor adhesion of the material being sputtered to the titanium substrate, and (2) no water vapor was released during the argon pure plasma vacuum environment that could cause impure elements in the deposition of the material. Once the tablets were dry and fixed for the vacuum coating process, they were placed in a PVD imbalanced magnetron.

The silver (Ag) content of the test items was 1.00 ± 0.2 mg/cm^2^, and the total silver content per test item was 5.0 ± 0.1 mg. Since the implantation of pylons in DSA patients is permanent, this coating specification was selected to combine the well-established bactericidal properties of silver [23] with a relatively fast dissolution of the silver layer, in order to avoid the toxic consequences of long-term exposure to silver [21]. Positive verification of this specification, patented in [16], was reported in our animal study [6], where the skin- and bone-integrated pylons (SBIPs) had a silver layer thin enough to dissolve within about 4–6 weeks after implantation. That period was sufficient for the skin to regenerate into the porous cladding of the SBIP and establish a sustainable natural barrier against infection.

The process of coating with TiN or ZrN requires similar parameters and gas combinations to coating with Ag. The power used to create a film was in the 5 kW range for a period of 1 h to build 1 micron of coating. Argon gas (non-reactive) formed the main plasma needed to create the energy required to atomize the target material. Nitrogen gas is the reactive gas that is the necessary catalyst to transform titanium or zirconium into a nitride film or coating.

For titanium nitride, we used a VT-3000 cathodic arc system with a deposit arc current set at 500 amps, argon gas at 600 sccm, and nitrogen at 600 sccm for 10 min, because a cathodic arc builds quicker than magneton sputtering. Negative bias was set at 100 volts.

For zirconium nitride, we used a VT-3000 cathodic arc system with a deposit arc current set at 500 amps and argon gas at 600 sccm for 10 min, because a cathodic arc builds quicker than magnetron sputtering. Negative bias was set at 100 volts.

For pure copper (Cu) coating, lower power was required to create a 1-micron film, due to copper’s propensity to self-sputter via the energy of the plasma transfer itself, and only argon (Ar) gas plasma was used for the deposition. Copper coating was targeted at location #5 in the Hayzer system via magnetron at 5 kW. We used only argon gas at 500 sccm for 15 min, and bias was set at 100 volts. The power used to create the film was in the 5 kW range for a period of 1 h to build 1 micron of coating. Argon gas (non-reactive) formed the main plasma required to create the energy needed to atomize the target material. Nitrogen gas is the reactive gas providing the necessary catalyst to transform titanium or zirconium into a nitride film or coating.

### 2.2. Cells

Fetal mesenchymal stem cells (FetMSCs) were obtained from a shared research facility’s “Vertebrate Cell Culture Collection” (supported by the Ministry of Science and Higher Education of the Russian Federation at the Institute of Cytology of the Russian Academy of Sciences (St. Petersburg, Russia)). FetMSCs were cultivated in DMEM supplemented with 10% fetal bovine serum (FBS), 1 mM Sodium Pyruvate, 6 mM L-glutamine, 4.5 g/L Glucose, 0.1 mM MEM Non-Essential Amino Acids, and antibiotics 1% penicillin-streptomycin (Gibco, Waltham, MA, USA). Human MG-63 cells were cultured in α-DMEM medium (Gibco, Waltham, MA, USA) supplemented with 10% FBS (Gibco, Waltham, MA, USA) and antibiotics 1% penicillin-streptomycin (Gibco, Waltham, MA, USA) at 37 °C and 5% CO_2_. For cell dissociation, 0.25% trypsin-EDTA (Gibco, Waltham, MA, USA) solution was employed at high cell confluence (≥90%). After 2–3 passages for increasing the number of cells, the cells were used for experimental procedures.

### 2.3. Scanning Electron Microscopy

To evaluate the formation of a cell monolayer on the surface of microporous titanium with different metal coatings using scanning electron microscopy (SEM), the samples were preliminarily autoclaved. Then, a cell suspension of either FetMSCs or MG-63 (5 × 10^6^/mL) was applied to the surface of the samples for 3 days in a CO_2_ incubator (Thermo Fisher Scientific, Waltham, MA, USA) at 37 °C. After that, a nutrient medium was added to each Petri dish, ensuring complete coverage of the implant surface. Upon completion of co-cultivation, the samples were washed with Dulbecco’s phosphate buffer (PBS) (Sigma-Aldrich, St. Louis, MO, USA) and fixed in a 2.5% glutaraldehyde solution in phosphate buffer (pH = 7.0, Sigma-Aldrich, St. Louis, MO, USA). Then, after washing, the samples were successively dehydrated for 30 min in 30, 50, 70, 90, and 96% absolute ethanol. Next, conductive silver coatings of approximately 10 nm thickness were applied using a Leica EM SCD500 microscope (Leica Microsystems, Wetzlar, Germany). The morphology of the cell monolayer was assessed using a Zeiss Auriga scanning electron microscope (Carl Zeiss, Oberkochen, Germany) in SE (secondary electron) modes.

### 2.4. Confocal Microscopy

The formation of the FetMSCs and MG-63 cell monolayer on the surface of microporous Ti disks was visualized employing the Olympus FV3000 confocal system (Olympus, Tokyo, Japan). Cells (0.1 × 10^6^/mL cells) were placed in Matrigel (0.2 mg/mL, Corning, New York, NY, USA) on the surface of Ti disks and kept overnight. After incubation, the cells were washed with PBS, stained with TMRM and LysoTracker, fixed in a 10% formalin solution (Sigma-Aldrich, St. Louis, MO, USA), and mounted using a mounting medium containing DAPI (Ibidi, Graefelfing, Germany). Unstained cells were used as controls.

### 2.5. Testing of Antibacterial Activities

The antimicrobial activity of the various materials for implants was tested on *Staphylococcus aureus* ATCC 29213. The disks were mounted onto the surface of LB-agar dishes and gently pressed with sterile tweezers to ensure uniform contact of the disk with the agar surface. Dishes were incubated at 37 °C for several days as indicated to allow diffusion of ions into the agar. Next, 1 mL of bacterial suspension with a density of 1.5 × 10^8^ CFU/mL was loaded onto the surface of the Petri dish with a LB-agar and evenly spread over the surface. Then, the remaining liquid was removed and dishes were dried. The Petri dishes were incubated at 37 °C for 24 h, and the diameter of the growth inhibition zones was measured.

### 2.6. Analysis of the Matrix Metalloproteinase Production

The activity of matrix metalloproteinases (MMP-1, MMP-2, and MMP-9) in the culture medium on day 1 following co-incubation with uncoated and differently coated titanium samples was determined using the zymography method described elsewhere [24]. Gelatin and casein were used as substrates to evaluate the activity of gelatinases (MMP-2, MMP-9) and collagenases (MMP-1, MMP-8), respectively. A gel (10% acrylamide) contained 1.0 mg/mL gelatin or 0.5 mg/mL casein. Two micrograms of protein per lane were loaded into the gel. The protein content in the probes was measured using a Bradford protein assay. After electrophoresis, the gel was washed twice with 2.5% Triton X-100 for 30 min and then incubated in a buffer solution (50 mM Tris-HCl pH 7.6, 0.15 M NaCl, 10 mM CaCl_2_, 0.05% Brij 35) for 12 h. Then, the gel was stained with Coomassie Blue R-250 (0.25% Coomassie brilliant blue R-250 in 40% isopropanol for 2 h) and, after destaining (with 7% acetic acid for 1 h), the bands containing MMPs were developed as non-stained bands. A medium conditioned by HT-1080 fibroblasts obtained from the Culture Collections of Institute of Cytology of the Russian Academy of Sciences [RAS], St Petersburg, Russia, was used as a marker to determine MMP zones. It contains both MMP-2 and MMP-9 [25,26]. Bands of MMP-1 and MMP-8 activity were verified by molecular weight markers and in preliminary experiments by antibodies. For the quantitative assay, gels were scanned, and images were processed with QuantiScan 3 software. MMP activity was normalized to protein concentration. MMP activity, presented in arbitrary units, was analyzed with the program QuantiScan which calculated the product of the number of colored pixels of the MMP band by the color intensity. The results of densitometry were presented in the form of histograms as mean values ± SEM.

### 2.7. Quantitative Real-Time PCR

Total RNA from human FetMSCs and MG-63 cells lines on each material (Ag, TiN, ZrN, Cu, and non-coated Ti) was extracted with the RUplus RNA isolation kit (Biolabmix, LLC, Moscow, Russia) according to the manufactures’ protocol. The isolated total RNA was quantified using a nanodrop spectrophotometer (Thermo Scientific, Waltham, MA, USA). cDNA synthesis was performed using the M-MuLV–RH First Strand cDNA Synthesis Kit (Biolabmix, LLC, Russia). The mixture of 3 μg RNA, 50 μM oligodT primer, and DEPC-treated water was carefully vortexed, and droplets were collected by centrifuging; then it was heated at 70 °C for 2–3 min in order to melt secondary structures and the tube was placed on ice. After this, the mixture was added to the reaction mix (5× RT buffer mix, 0.1 M DTT, 10 mM dNTPs mix, M-MuLV–RH revertase (100 u/μL)), and for first strand synthesis, incubated at 42 °C for 1 h with subsequent cooling on ice for 2–3 min. The reaction was stopped by heating the reaction solution at 70 °C for 10 min. The product of reverse transcription reaction was used directly for PCR amplification or stored at *−*70 °C. cDNA for GAPDH and actin was used as a control for calculating fold differences in RNA levels of FetMSCs and MG-63 cells cultivated on Ti and material covered Ti disks. mRNA relative quantities were obtained using the 2^*−*ΔΔCt^ method, and forward and reverse primers specific for tested genes were designed with Pubmed nucleotide design (Primer-BLAST) software version 1.0.1 for all tested genes (Table 1). The samples were evaluated in the Bio-Rad CFX96 real-time PCR system (Bio-Rad, Hercules, CA, USA) according to the manufacturer’s protocol. Cycling conditions were as follows: 95 °C for *−*5 min, followed by 45 cycles of 95 °C for 30 s, 60 °C for 30 s, and 72 °C for 30 s, and a final cycle of 72 °C for 2 min. A melt curve was generated by heating from 65 °C to 95 °C at ramps of 0.5 °C/s.

### 2.8. Statistics

For each of the metal coating conditions, including the control, and for both kinds of co-cultured cells (MG-63 and FetMSCs), 3 independent disks were studied, giving a total of 42 disks across 14 conditions. Means and standard deviations of [gene expression/MMPs] were computed for each condition, across each of the 4 studied time points (1, 3, 7, and 14 days after culturing). For the MG-63 and FetMSC cell groups, respectively, a one-way ANOVA tested the equality of means on the 14th day at the 0.05 level of significance. Post hoc Tukey–Cramer mean comparisons were used to identify the specific coating whose [gene expression/MMPs] were significantly different from the rest. For both cell cultures, the Cu coating’s [gene expression/MMPs] were significantly lower than those of all others (*p* < 0.01). One-way analysis with the Kruskal–Wallis test was used to analyze the differences. The software program used for the statistical analysis was Statistica Version 9.2. For all experiments, distinctions were regarded as statistically reliable at *p* < 0.05.

## 3. Results

### 3.1. Cytotoxicity Profile of the Titanium Metal Coatings

**Figure 3.**
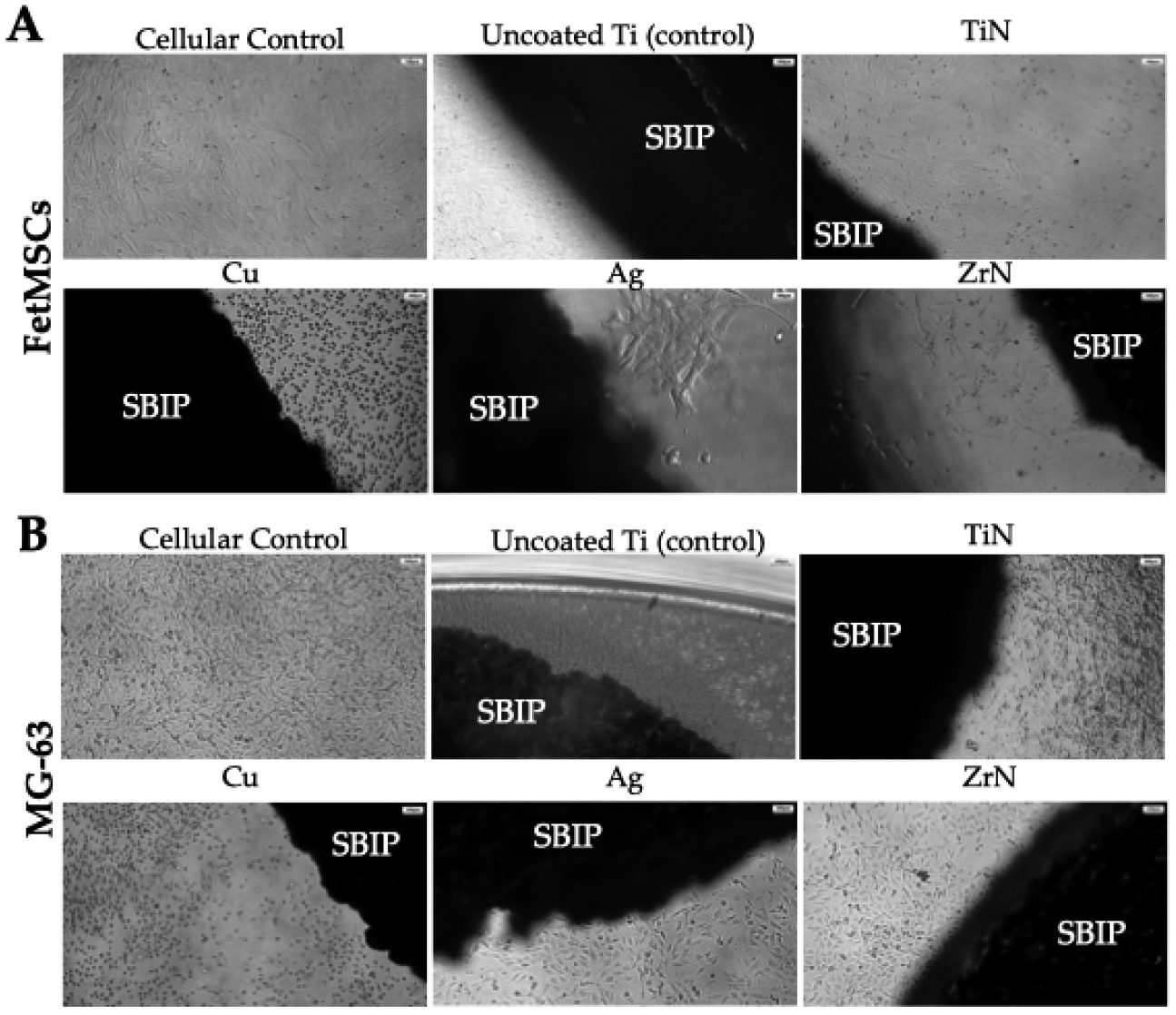
Light microscopy images of cells cultivated on the surface of the titan samples with various metal coatings. Cells were visualized using the inverted microscope Nikon Eclipse TS100 (Nikon, Tokyo, Japan). (**A**) Morphology of FetMSCs; (**B**) morphology of MG-63 cells. Note: Morphology of cells on the surface of a Petri dish was used as a control. SBIP—skin- and bone-integrated pylon composed of microporous titanium alloy. Scale bars, 100 μm.

To evaluate the cytotoxic properties of various metallic coatings of microporous titanium, FetMSCs and MG-63 cells were co-incubated with titanium disks for 1, 3, 7, and 14 days. Thus, when conducting light microscopy, after 24 h of co-cultivation, both cell cultures formed a monolayer directly on the surface of the implants and on the surface of the culture flask adjacent directly to the uncoated microporous titanium disk (Figure 3).

**Figure 4.**
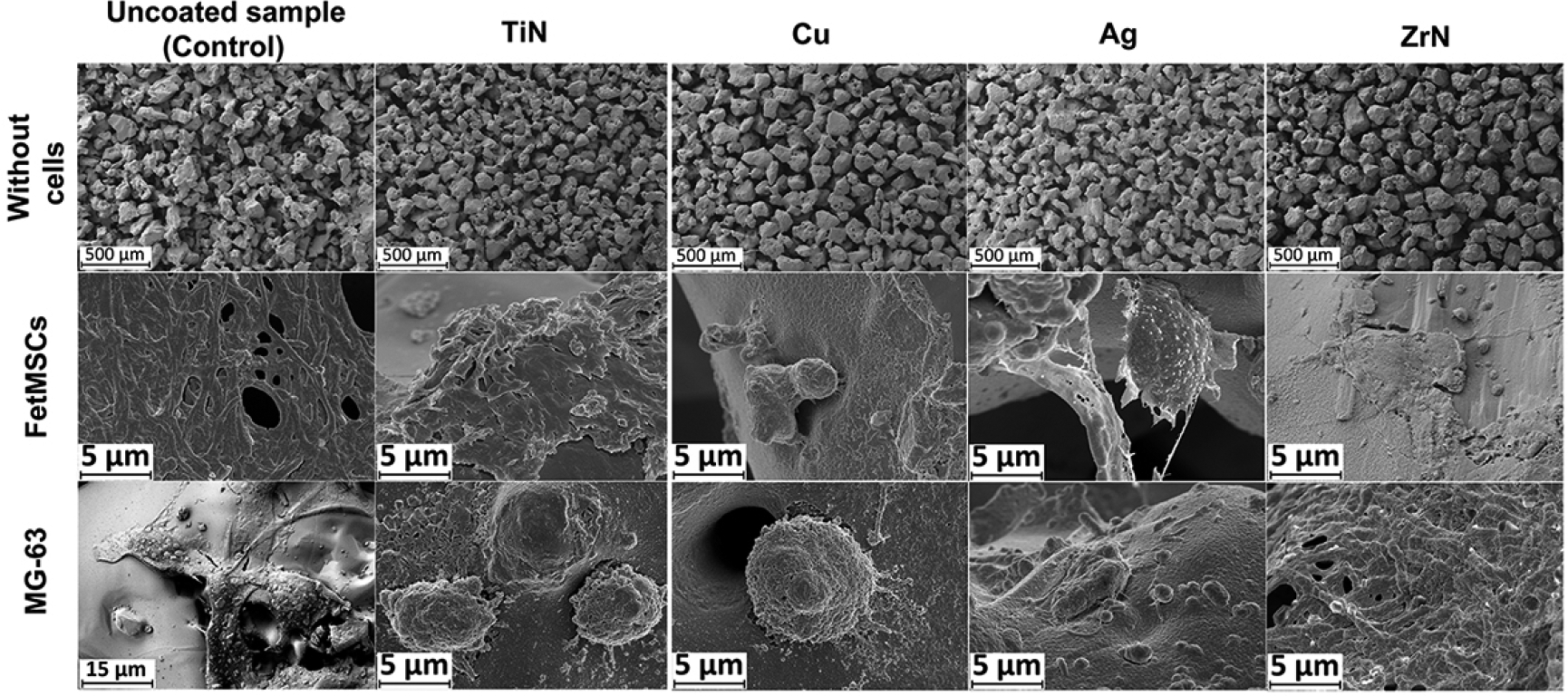
Scanning electron microcopy (SEM) studies of the microporous Ti disks with various metal coatings co-incubated with FetMSCs and MG-63 cells for 14 days.

The use of silver (Ag), titanium nitride (TiN), and zirconium nitride (ZrN) did not lead to a change in cell morphology, whereas when using a copper (Cu) coating, a significant change in cell morphology was observed, which became rounded with a violation of the formation of a cellular monolayer. The obtained data from light microscopy was further supported by the results of SEM studies, which also demonstrated the cytotoxicity of copper coatings towards FetMSCs and MG-63 cells (Figure 4).

**Figure 5.**
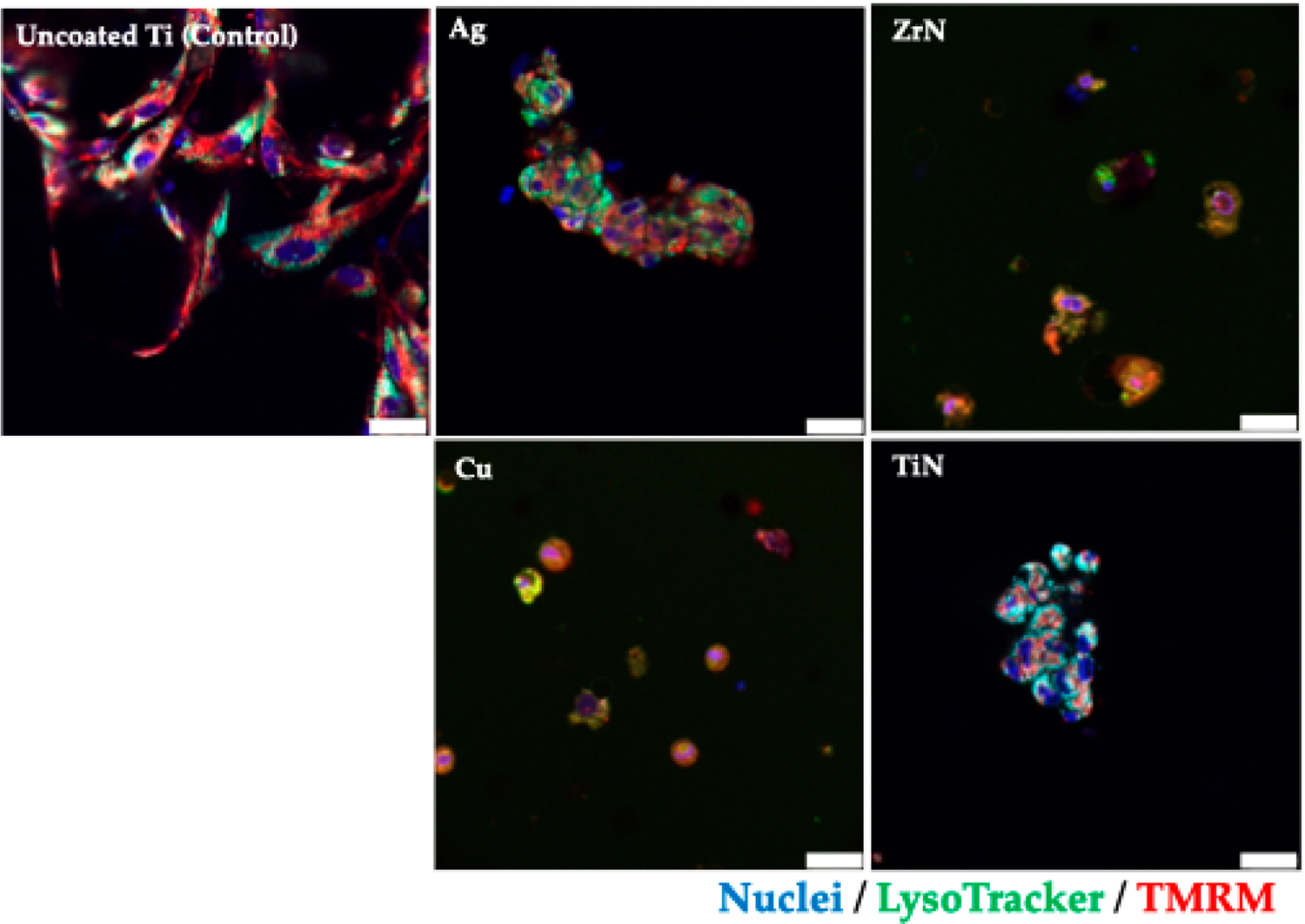
Confocal microscopy images of the FetMSCs co-cultured on the microporous Ti disks with various metal coatings for 14 days. Nuclei were stained with DAPI (blue), and mitochondria were stained with TMRM dye (red); lysosomes were detected with LysoTracker (green). Scale bars, 25 μm.

Furthermore, we assessed the FetMSC cell viability on Ti disks and also detected a cytotoxic influence of Cu coatings on the cells (Figure 5). To further identify viable cells, the latter were additionally stained with TMRM (*Tetramethylrhodamin-methylester)* dye, which identifies mitochondria in viable cells.

**Figure 6.**
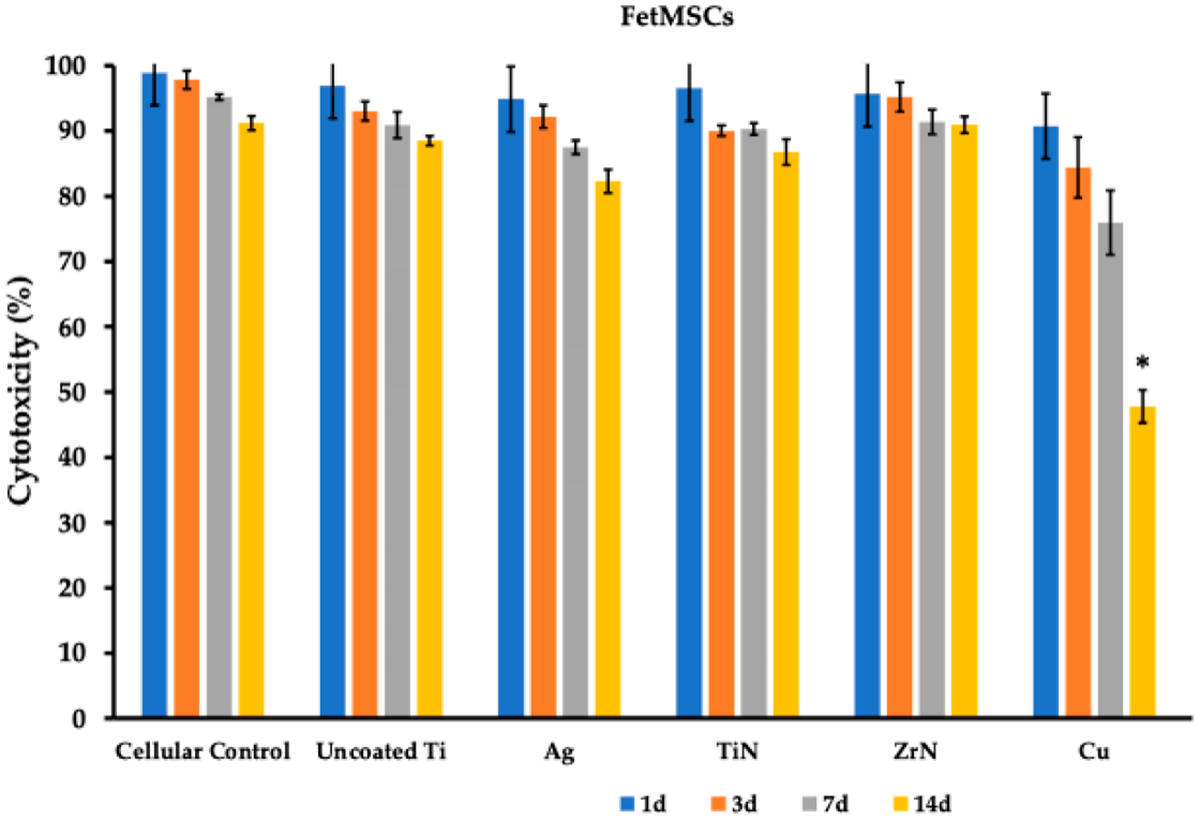
MTT assay of FetMSCs on microporous titanium disks with various metal coatings. Cell viability (%) was evaluated on the 1st, 3rd, 7th, and 14th day after co-incubation. Data is presented as mean (M) ± standard deviation (SD) from three independent experiments. *—statistically significant different (*p* < 0.01).

**Figure 7.**
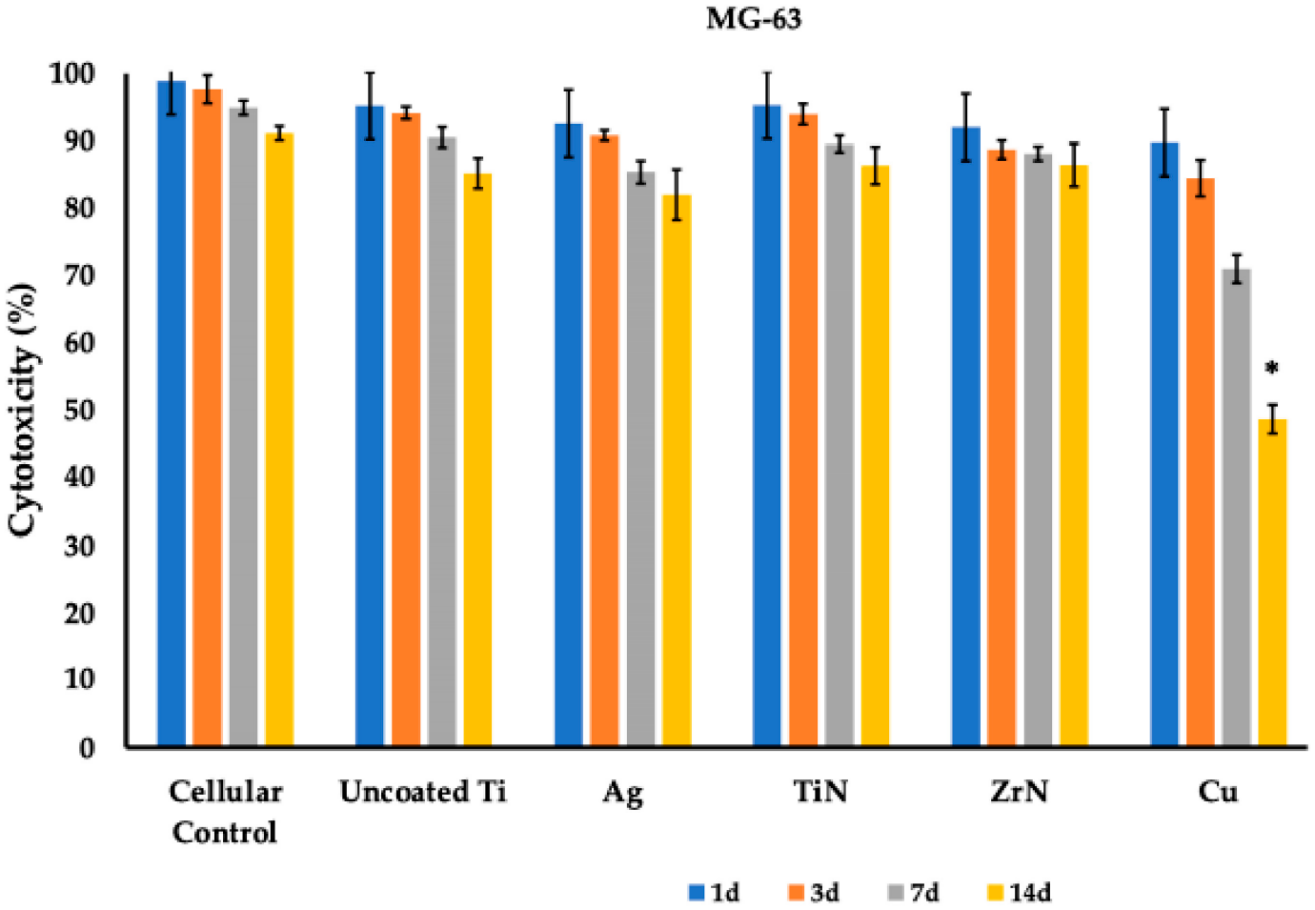
MTT assay of MG-63 cells on microporous titanium disks with various metal coatings. Cell viability (%) was evaluated on the 1st, 3rd, 7th, and 14th day after co-incubation. Data is presented as Mean (M) ± standard deviation (SD) from three independent experiments. *—statistically significant difference (*p* < 0.01).

When conducting the MTT test, it was found that application of the uncoated Ti disks led to a slight decrease in cell viability over the 14 days of co-incubation, which constituted 88.50 ± 0.72 % (FetMSCs) and 85.20 ± 2.25 % (MG-63) as compared to control cells cultivated on the surface of culture flasks—91.20 ± 1.08 % (FetMSCs) and 91.13 ± 1.06 % (MG-63) (Tables 2 and 3; Figures 6 and 7). When silver (Ag), titanium nitride (TiN), and zirconium nitride (ZrN) coatings were employed, we also did not detect significant toxicities during the follow-up period of 14 days, constituting 82.30 ± 1.78 %, 86.77 ± 1.96 %, 90.93 ± 1.27 %, and 88.00 ± 1.93 %, respectively, for FetMSCs and 82.00 ± 3.75 %, 86.33 ± 2.76 %, 86.43 ± 3.19 %, and 83.17 ± 5.28 %, respectively, for MG-63 cells (Tables 2 and 3; Figures 6 and 7). However, when copper coatings were employed, we detected significant cytotoxicity towards FetMSCs and MG-63 cells, which on day 14 constituted 47.80 ± 2.50 % and 48.77 ± 2.14 %, respectively (*p* < 0.001).

### 3.2. Antibacterial Properties of the Microporous Titanium Metal Coatings

**Figure 8.**
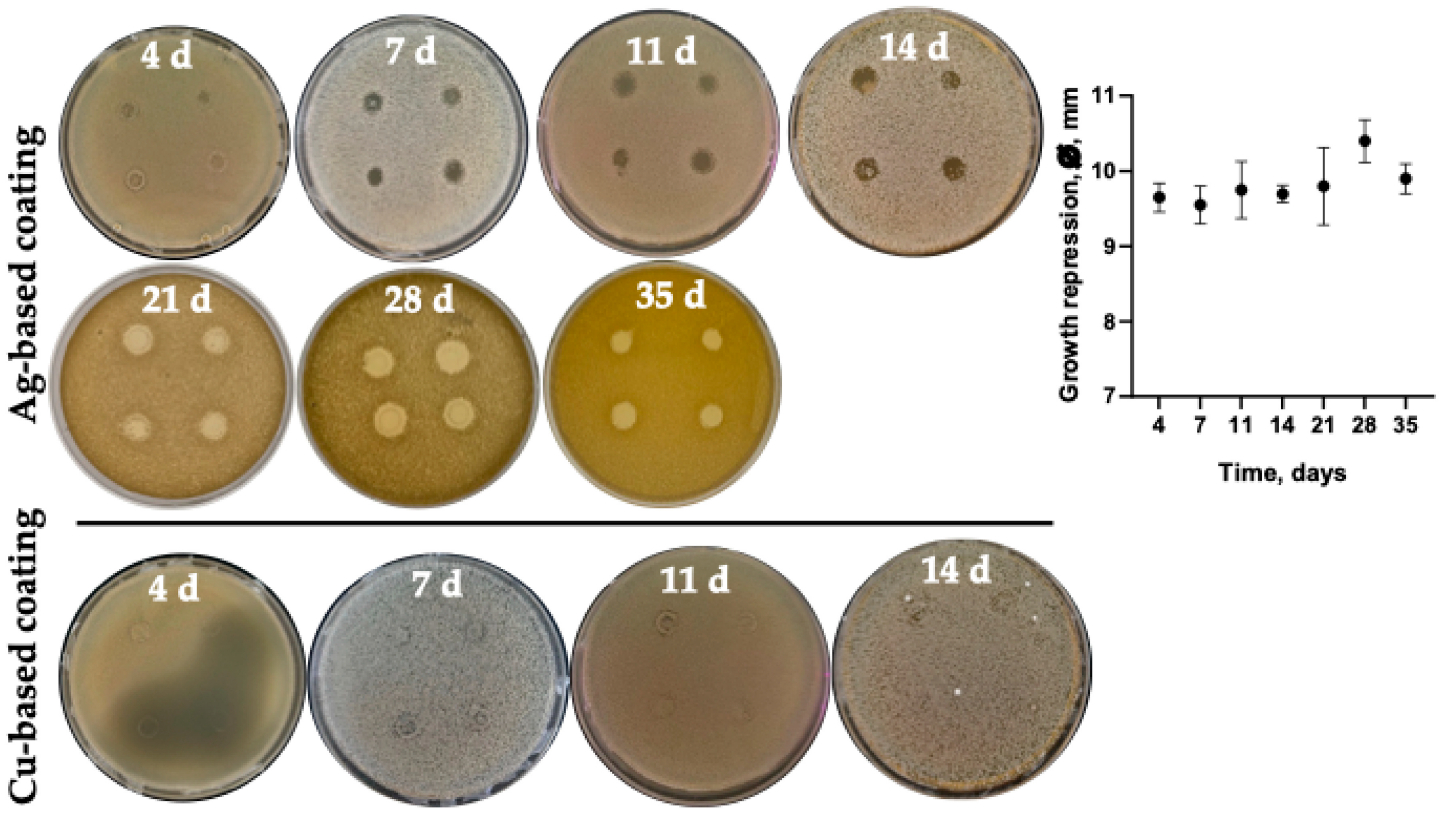
The growth repression zones of *S. aureus* on LB-agar after 3–4 days incubation with a mounted disk.

The antimicrobial activity of the microporous titanium disks with various metal coatings was evaluated on *S. aureus* ATCC 29213. Among all the Ti disks tested, a clear growth repression zone could be observed only for disks with silver (Ag) and copper (Cu). To evaluate whether the bacteriostatic effect will remain over a long period, the disks were mounted onto the surface of the agar in consequent series over 3–4 days. Although the antibacterial effect of Cu coatings was detected in the initial days of observation during the longer follow-up period, we did not observe any significant effect. The effect was stable and visible only for Ag coatings. Thus, as can be seen from Figure 8, the growth repression zone around the disk did not reduce over 35 days, suggesting a stable antimicrobial effect.

### 3.3. Induction of the Matrix Metalloproteinase Activity by Metal Coatings

**Figure 9.**
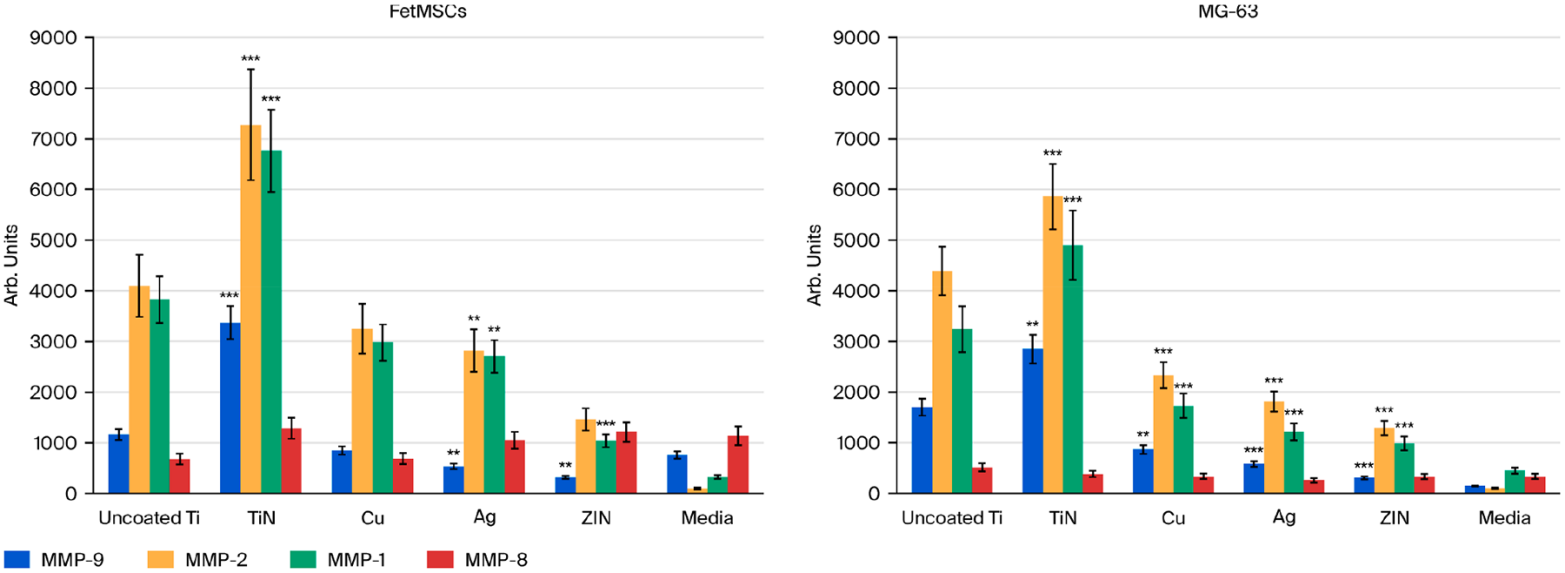
Analysis of matrix metalloproteinase activity (collagenases—MMP-1 and MMP-8; gelatinases—MMP-2 and MMP-9) in FetMSCs and MG-63 cells. **—statistically significant difference *p* < 0.01, ***—*p* < 0.001.

Considering the important role that matrix metalloproteinases play in bone tissue remodeling and the integration of titanium implants, we assessed enzyme activity during co-cultivation of cells with microporous titanium disks with various metal coatings (Figure 9; Tables 4 and 5). For FetMSCs, we detected a manifold increase in the levels of MMP-2, MMP-9, MMP-1, and MMP-8 on TiN coating relative to the uncoated control Ti sample, constituting 4742 ± 711 (MMP-2), 1616 ± 155 (MMP-9), 7280 ± 1092 (MMP-2), 3376 ± 324 (MMP-9), 6764 ± 812 (MMP-1), and 1287 ± 206 (MMP-8) for TiN-coated samples, while in the uncoated Ti sample (control), MMP levels were as follows: 4103 ± 615 (MMP-2), 1165 ± 112 (MMP-9), 3832 ± 460 (MMP-1), and 681 ± 109 (MMP-8) (*p* < 0.001). The levels of the same MMPs on copper (Cu) and silver (Ag) coatings were similar, and on the zirconium nitride coating, they were even lower as compared to the uncoated Ti disks. As a control (base), the measurement results for MMPs in cultivation media were shown. For MG-63 cells, we also observed the increase in the levels of all studied MMPs on TiN coatings as compared to the uncoated Ti sample—6419 ± 706 (MMP-2), 2560 ± 253 (MMP-9), 4575 ± 640 (MMP-1); 5860 ± 645 (MMP-2), 2846 ± 282 (MMP-9), 4899 ± 686 (MMP-1), and 384 ± 61 (MMP-8) (*p* < 0.001) for TiN coatings (*p* < 0.001). The levels of the same MMPs on copper, silver, and ZrN coatings were even lower as compared to the uncoated Ti control sample. As a control (base), the measurement results for MMPs in cultivation media were also shown.

### 3.4. Analysis of Focal Adhesion Markers of FetMSCs Cultured on Ti Disks with Various Metal Coatings

**Figure 10.**
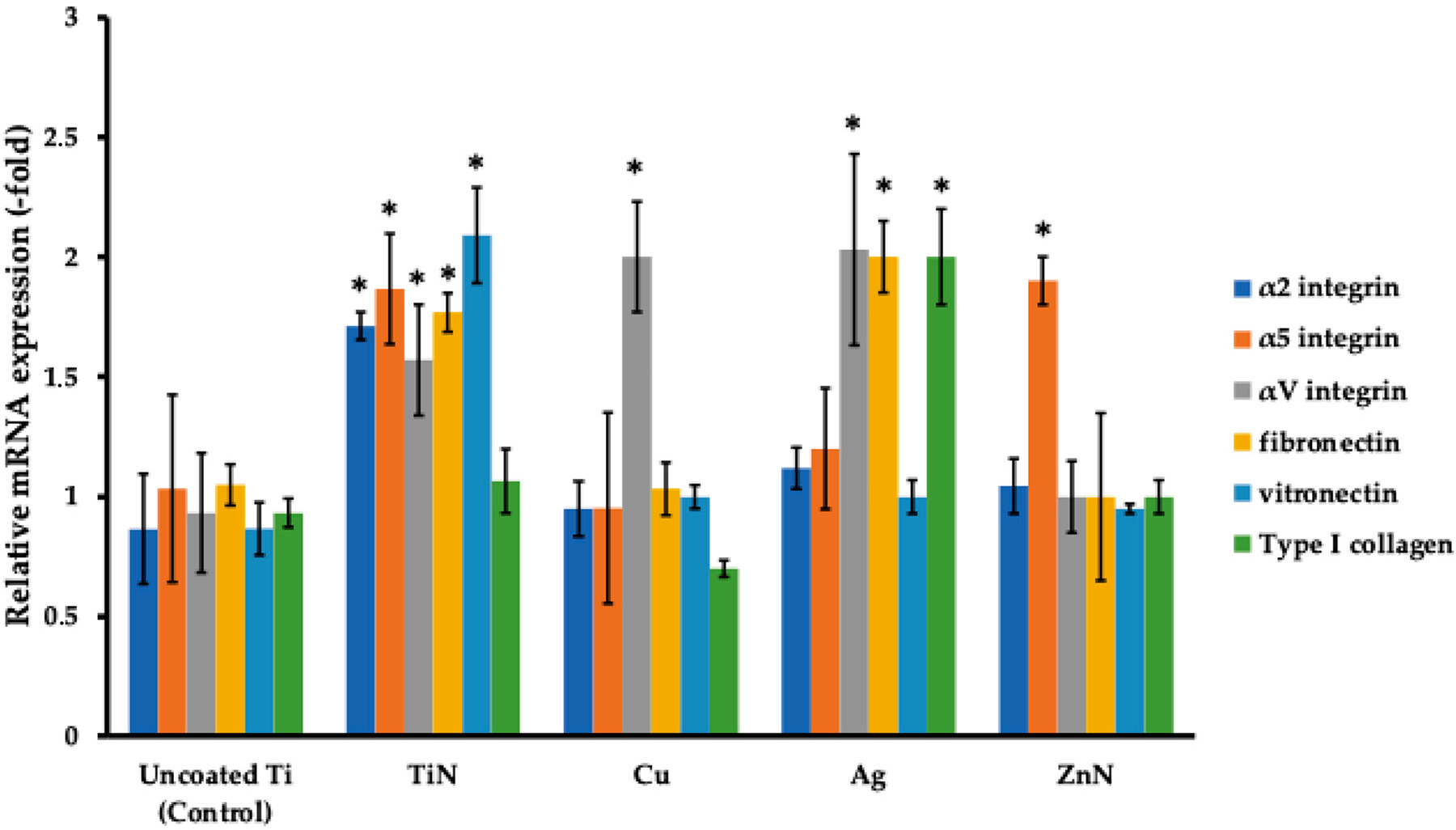
Comparison of expression of genes related for focal adhesion (α2 integrin, α5 integrin, αV integrin, type I collagen, fibronectin, and vitronectin) for FetMSCs co-incubated with microporous Ti disks with various metal coatings for 14 days. * *p* < 0.05 for testing mean expression levels.

Gene expression of α2 integrin (collagen-specific), α5 integrin (fibronectin-specific), αV integrin (vitronectin-specific), type I collagen, fibronectin, and vitronectin genes were assessed following 72 h of co-incubation on microporous Ti disks with various metal coatings (Figure 10). Thus, after 72 h of co-incubation for uncoated Ti samples, the values of the genes constituted 0.866 ± 0.23 (α2), 1.033 ± 0.058 (α5), 0.933 ± 0.115 (αV), 1.05 ± 0.087 (fibronectin), 0.8667 ± 0.115 (vitronectin), and 0.933 ± 0.1 (type I collagen). Intriguingly, when silver (Ag) and titanium nitride (TiN) coatings were employed, we detected a significant increase in the expression of the studied genes (Figure 10).

### 3.5. Analysis of Osteogenic Markers of MG-63 Cells Cultured on Ti Disks with Various Metal Coatings

**Figure 11.**
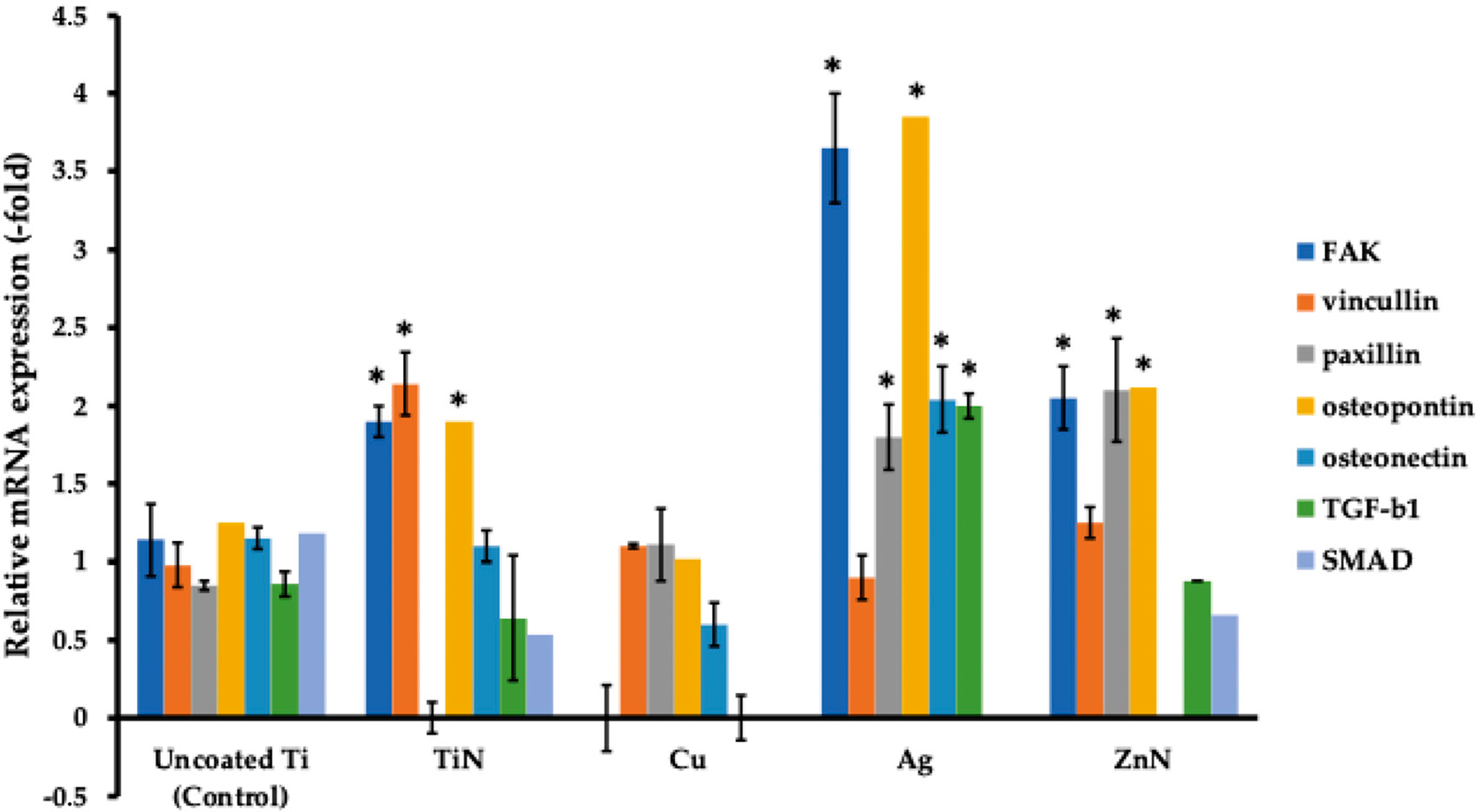
Comparison of expression of genes related to osteogenic markers (osteopontin, osteonectin, TGF-β1, SMAD) and genes related to focal adhesion (FAK, vinculin, paxillin) for MG-63 cells co-incubated with microporous Ti disks with various metal coatings for 14 days. * *p* < 0.05 for testing mean expression levels.

At the second stage, we evaluated the genes related to focal adhesion (FAK, vinculin, paxillin) and those related to osteogenic markers (osteopontin, osteonectin, TGF-β1, SMAD) for MG-63 cells after 14 days of cell co-incubation on Ti disks (Figure 11). Thus, for uncoated microporous Ti disks, the levels of expression of the studied genes constituted −1.14 ± 0.24 (FAK), 0.98 ± 0.1 (vinculin), 0.85 ± 0.21 (paxillin), 1.25 ± 0.35 (osteopontin), 1.15 ± 0.2 (osteonectin), 0.86 ± 0.196 (TGF-β1), and 1.18 ± 0.25 (SMAD). When silver (Ag), titanium nitride (TiN), and zirconium nitride (ZrN) coatings were employed, we detected an increase in the studied genes’ expressions, with the highest values for the silver coating—3.65 ± 0.07 (FAK), 0.9 ± 0.1 (vinculin), 1.8 ± 0.14 (paxillin), 3.85 ± 0.21 (osteopontin), 2.04 ± 0.06 (osteonectin), 2.0 ± 0.283 (TGF-β1), and 0 ± 0.21 (SMAD).

## 4. Discussion

The use of metal coatings on dental and orthopedic titanium implants is an important area in modern translational research, since these coatings may exert antibacterial properties, promote osseointegration, which is important for the implant’s mechanical stability, and facilitate soft tissue attachment [27–29].

The obtained data on the antibacterial activity of metal coatings of titanium disks (Figure 8), which showed the best activity for copper and silver, correspond to data presented in [23,30–32]. Although the silver coating reduced cell viability as compared to non-treated microporous titanium disks, it did not impair the cell adhesion and differentiation [33]. At the same time, use of the copper coating led to a significant increase in the cytotoxic effect of copper ions on both bone tissue cells and FetMSCs, leading to a noticeable change in cellular morphology and cell migration into the pores of the implant (Figures 6 and 7; Tables 2 and 3). Indeed, copper has been shown to have pronounced toxic activity against normal cells of the body, which significantly reduces the possibility of its clinical use. Presumably, the use of nanoformulation of copper composites will reduce the toxic effect [34,35].

To assess the processes of integration with soft and bone tissues, we assessed the expression of matrix metalloproteinase enzymes in vitro. Titanium implants do induce the expression of MMPs during the process of osseointegration [36–38]. Intriguingly, when compared to various metal coatings, the titanium nitride (TiN) coating induced the highest expression of MMPs in FetMSCs and MG-63 cells (Figure 9). Indeed, as shown by Oliva et al. [39], TiN-coated titanium may modulate inflammation through the inhibition of the TLR4/MyD88/NF-κB p65/NLRP3 pathway and induce extracellular matrix apposition. In parallel to the enhanced activity of MMPs, we also observed and increased mRNA production of genes related to focal cell adhesion (including FAK, vinculin, α2, α5, αV integrins, type I collagen, fibronectin, and vitronectin) in FetMSCs and MG-63 cells (Figure 9). The obtained data is in line with published results showing that TiN coatings also induced in vitro cellular responses with high cell adhesion molecule expression [40,41]. As was shown by Zreiqat et al., interaction between osteoblastic cells and biomaterials results in enhanced activation of Shc and the RAS/RAF/MAPK signaling pathway, as well as upregulation of c-fos signaling pathway [42].

Herein, we performed a comparative study of the most commonly used metal coatings in regard to their bioactive properties. To further increase their biointegrative properties, these coatings can be applied in combination with other bioactive molecules (including growth factors, peptides, etc.) [43–48]. Thus, in the recent study by Jiang et al., it was shown that incorporation of chitosan microspheres loaded with Bone Morphogenetic Protein-2 (BMP-2) and Platelet-Derived Growth Factor-BB (PDGF-BB) into the implant significantly enhanced osteogenic differentiation [49]. Another direction for possible research may be based on the site-selective metal coatings on the surface of the implant with various coatings on the bone-contacting surface and the skin-penetrating part of the DSA implant. Indeed, this approach showed efficacy for orthopedic titanium implants in preclinical studies [27].

## 5. Conclusions

When choosing the optimal microporous titanium coating for orthopedics, parameters such as antibacterial properties, cytotoxicity, and the induction of biointegration processes with soft and bone tissues should be considered. According to the results of this study, which assessed the mRNA expression of genes associated with the processes of osteogenesis and cell adhesion and studied the activity of matrix metalloproteinases, a silver coating is recommended. If the risk of infection is minimal and does not pose a threat to biointegration with surrounding tissues, the titanium nitride (TiN) coating can be recommended. At the same time, it is worth noting that a comparative analysis of titanium metal coatings was carried out only in vitro, which indicates the need for further preclinical assessments in vivo.

## Figures and Tables

**Figure 2.**
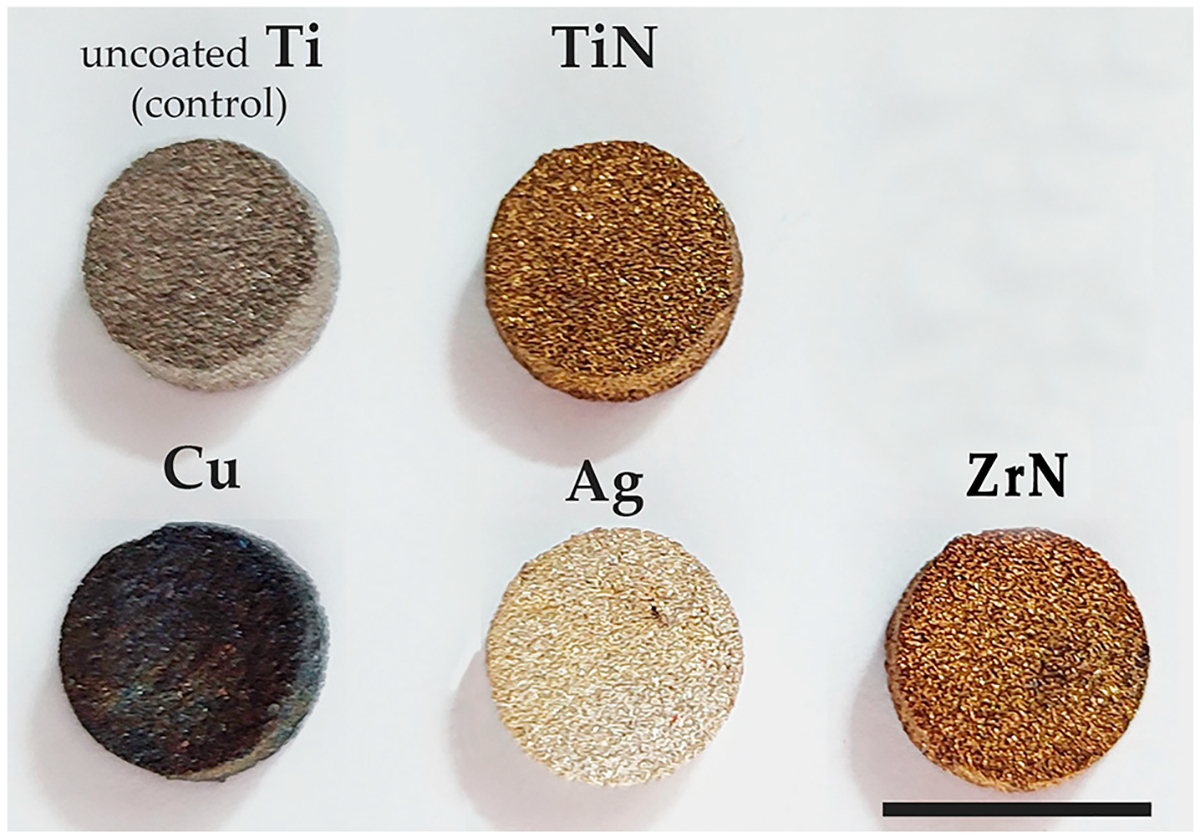
Coated samples: uncoated titanium medical grade; coating with titanium nitride (TiN); coating with silver (Ag); coating with zirconium nitride (ZrN); coating with copper. Scale bar, 1 cm.

**Table 1.** Forward and reverse primers specific for analyzed genes used for RT-PCR studies.

Gene	Primers (5′–3′)
Fibronectin	F: CAGCCTCTGGTTCAGACTGC
	R: TCTTGTCCTACATTCGGCGG
Vitronectin	F: TACCCCAAGCTCATCCGAGA
	R: ACTGTAGCTATGGGCAGGGA
Type I collagen	F: GGTGTAAGCHCTGGTGGTTA
	R: CCAGTTCTTGGCTGGGATGT
α2 integrin	F: GGCTGGCCCAGAGTTTACAT
	R: ATCGAAAAATCTCCTAACTT
α5 integrin	F: TTCAACTTAGACGCGGAGGC
	R: ATCGCCCCCTCTCCTAACTT
αV integrin	F: CCTAGGCACCCTCCTTCTGA
	R: TCACATTTGAGGACCTGCCC
FAK	F: GTCGTCTGCCTTCGCTTCA
	R: AGCAGGCCACATGCTTTACT
Paxillin	F: AAAGTTGCGGGGCATAGACG
	R: CAAGAACACAGGCCGTTTGG
Vinculin	F: GAGCAAAACCATCTCCCCGA
	R: CTGCCTCAGCTACAACACCT
Osteopontin	F: CAGCAGCAGCAGGAGGAG
	R: ACGGCTGTCCCAATCAGAAG
Osteonectin	F: TCGGCATCAAGCAGAGGAAT
	R: GTCCCTAGAGCCCCTGAGAA
TGF-β1	F: TGTCCAGGCAAGAAATGGCA
	R: AGGAACCGCAGCACTCATAC
SMAD4	F: ATGCTCAGTGGCTTCTCGAC
	R: CCTAGGGGAGAGCAGGAAGG

**Table 2.** Means and (standard deviations) of FetMSCs across different materials at different time points.

	Cellular Control	Uncoated Ti (Control)	Ag	TiN	ZrN	Cu
**1d**	98.93 (0.47)	96.93 (1.27)	94.87 (2.90)	96.53 (1.42)	95.67 (4.56)	90.73 (1.43)
**3d**	97.83 (1.40)	93.03 (1.46)	92.20 (1.73)	90.03 (0.81)	95.20 (2.23)	84.40 (4.63)
**7d**	95.17 (0.45)	90.90 (2.02)	87.50 (1.06)	90.30 (0.90)	91.40 (1.90)	75.97 (4.94)
**14d**	91.20 (1.08)	88.50 (0.72)	82.30 (1.78)	86.77 (1.96)	90.93 (1.27)	47.80 (2.50)

**Table 3.** Means and (standard deviations) of MG-63 across different materials at different time points.

	Cellular Control	Uncoated Ti (Control)	Ag	TiN	ZrN	Cu
**1d**	98.93 (0.55)	95.20 (1.06)	92.60 (2.17)	95.33 (4.31)	92.03 (2.92)	89.73 (0.45)
**3d**	97.67 (2.10)	94.17 (0.91)	90.80 (0.78)	93.97 (1.53)	88.70 (1.37)	84.47 (2.71)
**7d**	94.93 (1.05)	90.53 (1.53)	85.37 (1.68)	89.53 (1.27)	88.10 (1.06)	71.03 (2.10)
**14d**	91.13 (1.06)	85.20 (2.25)	82.00 (3.75)	86.33 (2.76)	86.43 (3.19)	48.77 (2.14)

**Table 4.** Analysis of matrix metalloproteinase activity (collagenases—MMP-1 and MMP-8; gelatinases—MMP-2 and MMP-9) in FetMSCs. Data is presented as mean (M) ± standard error of the mean (SEM) from three independent experiments.

Samples	MMP-9		MMP-2		MMP-1		MMP-1	
	Mean	SEM	Mean	SEM	Mean	SEM	Mean	SEM
**Media**	762	73	100	15	328	39	1140	182
**Uncoated Ti (Control)**	1165	112	4103	615	3832	460	681	109
**TiN**	3376	324	7280	1092	6764	812	1287	206
**Cu**	852	82	3253	488	2981	358	693	111
**Ag**	539	52	2821	423	2704	325	1050	168
**ZrN**	321	31	1464	220	1040	125	1215	194

**Table 5.** Analysis of matrix metalloproteinase activity (collagenases—MMP-1 and MMP-8; gelatinases—MMP-2 and MMP-9) in MG-63 cells. Data is presented as mean (M) ± standard error of the mean (SEM) from three independent experiments.

Samples	MMP-9		MMP-2		MMP-1		MMP-1	
	Mean	SEM	Mean	SEM	Mean	SEM	Mean	SEM
**Media**	150	15	100	11	451	63	336	54
**Uncoated Ti (Control)**	1705	169	4393	483	3244	454	513	82
**TiN**	2846	282	5860	645	4899	686	384	61
**Cu**	869	86	2335	257	1729	242	337	54
**Ag**	583	58	1815	200	1215	170	261	42
**ZrN**	307	30	1292	142	987	138	331	53

## Data Availability

The datasets used and/or analyzed during the current study are available from the corresponding authors, Maxim Shevtsov and Mark Pitkin, on reasonable request.

## Abbreviations

BMP: bone mineral density
CaP: calcium phosphate
DSA: direct skeletal attachment
FAK: focal adhesion kinase
FetMSCs: fetal mesenchymal stem cells
HA: hydroxyapatite
ML-ALD: molecular layering of atomic layer deposition
MMPs: Matrix Metalloproteinases
MTT: 3-(4,5-di methyl thiazol-2-yl)-2,5-diphenyltetrazolium bromide
PDGF-BB: Platelet-Derived Growth Factor-BB
RT-PCR: Real-Time Polymerase Chain Reaction
RTQ: removal torque tests
SBIP: skin- and bone-integrated pylon
SEM: scanning electron microscopy
SLM: selective laser melting
SMAD4: SMAD family member 4, Mothers against decapentaplegic homolog 4
TGF-β1: Transforming growth factor beta
TiN: Titanium Nitride
TMRM: Tetramethylrhodamine-methylester
ZrN: Zirconium Nitride

## References

[1] Farina D; Vujaklija I; Brånemark R; Bull AMJ; Dietl H; Graimann B; Hargrove LJ; Hoffmann KP; Huang HH; Ingvarsson T; Toward higher-performance bionic limbs for wider clinical use. Nat. Biomed. Eng 2023, 7, 473–485. 34059810 10.1038/s41551-021-00732-x

[2] Day Y; Thompson AR; Andaya VR; Smith K; Doung YC; Gundle KR; Hayden JB; Tran TH; Mohler DG; Avedian RS; Survival of Proximal Tibial Endoprostheses Using Compressive Osseointegration: A Multi-Institution Retrospective Study. J. Surg. Oncol 2025, 132, 284–293. 40525763 10.1002/jso.70013

[3] Groundland J; Brown JM; Monument M; Bernthal N; Jones KB; Randall RL What Are the Long-term Surgical Outcomes of Compressive Endoprosthetic Osseointegration of the Femur with a Minimum 10-year Follow-up Period? Clin. Orthop. Relat. Res 2022, 480, 539–548. 34559734 10.1097/CORR.0000000000001979 PMC8846358

[4] Fearing BV; Gitajn IL; Romereim SM; Hoellwarth JS; Wenke JC Basic science review of transcutaneous osseointegration: Current status, research gaps and needs, and defining future directions. OTA Int. 2025, 8, e367. 40071166 10.1097/OI9.0000000000000367 PMC11892713

[5] Shrivas S; Samaur H; Yadav V; Boda SK Soft and Hard Tissue Integration around Percutaneous Bone-Anchored Titanium Prostheses: Toward Achieving Holistic Biointegration. ACS Biomater. Sci. Eng 2024, 10, 1966–1987. 38530973 10.1021/acsbiomaterials.3c01555

[6] Shevtsov M; Gavrilov D; Yudintceva N; Zemtsova E; Arbenin A; Smirnov V; Voronkina I; Adamova P; Blinova M; Mikhailova N; Protecting the skin-implant interface with transcutaneous silver-coated skin-and-bone-intergrated-pylon (SBIP) in pig and rabbit dorsum models. J. Biomed. Mater. Res. B Appl. Biomater 2021, 109, 584–595. 32935912 10.1002/jbm.b.34725 PMC9317244

[7] Shevtsov M; Pitkin E; Combs SE; Meulen GV; Preucil C; Pitkin M Comparison In Vitro Study on the Interface between Skin and Bone Cell Cultures and Microporous Titanium Samples Manufactured with 3D Printing Technology Versus Sintered Samples. Nanomaterials 2024, 14, 1484. 39330641 10.3390/nano14181484 PMC11434446

[8] Van den Borre CE; Zigterman BGR; Mommaerts MY; Braem A How surface coatings on titanium implants affect keratinized tissue: A systematic review. J. Biomed. Mater. Res. B Appl. Biomater 2022, 110, 1713–1723. 35103386 10.1002/jbm.b.35025 PMC9306745

[9] Braem A; Chaudhari A; Vivan Cardoso M; Schrooten J; Duyck J; Vleugels J Peri- and intra-implant bone response to microporous Ti coatings with surface modification. Acta Biomater. 2014, 10, 986–995. 24161385 10.1016/j.actbio.2013.10.017

[10] Xue T; Attarilar S; Liu S; Liu J; Song X; Li L; Zhao B; Tang Y Surface Modification Techniques of Titanium and its Alloys to Functionally Optimize Their Biomedical Properties: Thematic Review. Front. Bioeng. Biotechnol 2020, 8, 603072. 33262980 10.3389/fbioe.2020.603072 PMC7686851

[11] Han X; Ma J; Tian A; Wang Y; Li Y; Dong B; Tong X; Ma X Surface modification techniques of titanium and titanium alloys for biomedical orthopaedics applications: A review. Colloids Surf. B Biointerfaces 2023, 227, 113339. 37182380 10.1016/j.colsurfb.2023.113339

[12] Prachar P; Bartakova S; Brezina V; Cvrcek L; Vanek J Cytocompatibility of implants coated with titanium nitride and zirconium nitride. Bratisl. Med. J 2015, 116, 154–156. 10.4149/bll_2015_031 25869562

[13] van Hove RP; Sierevelt IN; van Royen BJ; Nolte PA Titanium-Nitride Coating of Orthopaedic Implants: A Review of the Literature. Biomed. Res. Int 2015, 2015, 485975. 26583113 10.1155/2015/485975 PMC4637053

[14] Das M; Bhattacharya K; Dittrick SA; Mandal C; Balla VK; Sampath Kumar TS; Bandyopadhyay A; Manna I In situ synthesized TiB-TiN reinforced Ti6Al4V alloy composite coatings: Microstructure, tribological and in-vitro biocompatibility. J. Mech. Behav. Biomed. Mater 2014, 29, 259–271. 24121827 10.1016/j.jmbbm.2013.09.006

[15] Hempel F; Finke B; Zietz C; Bader R; Weltmann KD; Polak M Antimicrobial surface modification of titanium substrates by means of plasma immersion ion implantation and deposition of copper. Surf. Coat. Technol 2014, 256, 52–58.

[16] Pitkin M; Raykhtsaum G Skin Integrated Device. US Patent 8257435, 4 September 2012. Available online: http://www.google.com/patents/US8257435 (accessed on 9 August 2025).

[17] Ching HA; Choudhury D; Nine MJ; Abu Osman NA Effects of surface coating on reducing friction and wear of orthopaedic implants. Sci. Technol. Adv. Mater 2014, 15, 014402. 27877638 10.1088/1468-6996/15/1/014402 PMC5090599

[18] Galetz MC; Fleischmann EW; Konrad CH; Schuetz A; Glatzel U Abrasion resistance of oxidized zirconium in comparison with CoCrMo and titanium nitride coatings for artificial knee joints. J. Biomed. Mater. Res. B Appl. Biomater 2010, 93, 244–251. 20162723 10.1002/jbm.b.31581

[19] Galetz MC; Seiferth SH; Theile B; Glatzel U Potential for adhesive wear in friction couples of UHMWPE running against oxidized zirconium, titanium nitride coatings, and cobalt-chromium alloys. J. Biomed. Mater. Res. B Appl. Biomater 2010, 93, 468–475. 20186822 10.1002/jbm.b.31604

[20] Lee D-W; Lee H-S; Park J-H; Shin S-M; Wang J-P Sintering of titanium hydride powder compaction. Procedia Manuf. 2015, 2, 550–557.

[21] Hadrup N; Sharma AK; Jacobsen NR; Loeschner K Distribution, metabolism, excretion, and toxicity of implanted silver: A review. Drug Chem. Toxicol 2022, 45, 2388–2397. 34455878 10.1080/01480545.2021.1950167

[22] SreeHarsha KS Principles of Physical Vapor Deposition of Thin Films, 1st ed.; Elsevier: Amsterdam, The Netherlands; Boston, MA, USA, 2006; pp. xi, 1160p.

[23] Liang J; Chen A; Wu M; Tang X; Feng H; Liu J; Xie G A shellfish-inspired bionic microstructure design for biological implants: Enhancing protection of antibacterial silver-loaded coatings and promoting osseointegration. J. Mech. Behav. Biomed. Mater 2025, 167, 106963. 40120143 10.1016/j.jmbbm.2025.106963

[24] Oliver GW; Stetler-Stevenson WG; Kleiner DE Zymography, Casein Zymography, and Reverse Zymography: Activity Assays for Proteases and their Inhibitors. In Proteolytic Enzymes: Tools and Targets; Sterchi EE, Stöcker W, Eds.; Springer: Berlin/Heidelberg, Germany, 1999; pp. 63–76.

[25] Toth M; Sohail A; Fridman R Assessment of gelatinases (MMP-2 and MMP-9) by gelatin zymography. Methods Mol. Biol 2012, 878, 121–135. 22674130 10.1007/978-1-61779-854-2_8

[26] Roomi MW; Kalinovsky T; Monterrey J; Rath M; Niedzwiecki A In vitro modulation of MMP-2 and MMP-9 in adult human sarcoma cell lines by cytokines, inducers and inhibitors. Int. J. Oncol 2013, 43, 1787–1798. 24085323 10.3892/ijo.2013.2113 PMC3834263

[27] Tang W; Fischer NG; Kong X; Sang T; Ye Z Hybrid coatings on dental and orthopedic titanium implants: Current advances and challenges. BMEMat 2024, 2, e12105.

[28] Gkioka M; Rausch-Fan X Antimicrobial Effects of Metal Coatings or Physical, Chemical Modifications of Titanium Dental Implant Surfaces for Prevention of Peri-Implantitis: A Systematic Review of In Vivo Studies. Antibiotics 2024, 13, 908. 39335082 10.3390/antibiotics13090908 PMC11428254

[29] Amirtharaj Mosas KK; Chandrasekar AR; Dasan A; Pakseresht A; Galusek D Recent Advancements in Materials and Coatings for Biomedical Implants. Gels 2022, 8, 323. 35621621 10.3390/gels8050323 PMC9140433

[30] Li J; Jiang B; Yang L; Zhang P; Wu J; Yang Y; Yang Y; Wang G; Chen J; Zhang L; Dual-functional titanium implants via polydopamine-mediated lithium and copper co-incorporation: Synergistic enhancement of osseointegration and antibacterial efficacy. Front. Bioeng. Biotechnol 2025, 13, 1593545. 40421118 10.3389/fbioe.2025.1593545 PMC12104301

[31] Yang J; Qin H; Chai Y; Zhang P; Chen Y; Yang K; Qin M; Zhang Y; Xia H; Ren L; Molecular mechanisms of osteogenesis and antibacterial activity of Cu-bearing Ti alloy in a bone defect model with infection in vivo. J. Orthop. Transl 2021, 27, 77–89. 10.1016/j.jot.2020.10.004 PMC7779545 33437640

[32] Croes M; Bakhshandeh S; van Hengel IAJ; Lietaert K; van Kessel KPM; Pouran B; van der Wal BCH; Vogely HC; Van Hecke W; Fluit AC; Antibacterial and immunogenic behavior of silver coatings on additively manufactured porous titanium. Acta Biomater. 2018, 81, 315–327. 30268917 10.1016/j.actbio.2018.09.051

[33] Shevtsov M; Pitkin E; Combs SE; Yudintceva N; Nazarov D; Meulen GV; Preucil C; Akkaoui M; Pitkin M Biocompatibility Analysis of the Silver-Coated Microporous Titanium Implants Manufactured with 3D-Printing Technology. Nanomaterials 2024, 14, 1876. 39683264 10.3390/nano14231876 PMC11643975

[34] Tsymbal S; Li G; Agadzhanian N; Sun Y; Zhang J; Dukhinova M; Fedorov V; Shevtsov M Recent Advances in Copper-Based Organic Complexes and Nanoparticles for Tumor Theranostics. Molecules 2022, 27, 7066. 36296659 10.3390/molecules27207066 PMC9611640

[35] Osman H; Tang X; Wei Q; Liu B; Gao J; Wang Y In Situ Electrochemical Fabrication of Photoreactive Ag-Cu Bimetallic Nanocomposite Coating and Its Antibacterial-Osteogenic Synergy. ACS Appl. Bio Mater 2025, 8, 6326–6338. 10.1021/acsabm.5c00802 40526488

[36] Shubayev VI; Brånemark R; Steinauer J; Myers RR Titanium implants induce expression of matrix metalloproteinases in bone during osseointegration. J. Rehabil. Res. Dev 2004, 41, 757–766. 15685464 10.1682/jrrd.2003.07.0107

[37] Oum’hamed Z; Garnotel R; Josset Y; Trenteseaux C; Laurent-Maquin D Matrix metalloproteinases MMP-2, -9 and tissue inhibitors TIMP-1, -2 expression and secretion by primary human osteoblast cells in response to titanium, zirconia, and alumina ceramics. J. Biomed. Mater. Res. A 2004, 68, 114–122. 14661256 10.1002/jbm.a.20001

[38] Fu C; Xie J; Hu N; Liang X; Chen R; Wang C; Chen C; Xu C; Huang W; Paul Sung KL Titanium particles up-regulate the activity of matrix metalloproteinase-2 in human synovial cells. Int. Orthop 2014, 38, 1091–1098. 24271334 10.1007/s00264-013-2190-0 PMC3997776

[39] Oliva S; Diomede F; Della Rocca Y; Mazzone A; Marconi GD; Pizzicannella J; Trubiani O; Murmura G Anti-TLR4 biological response to titanium nitride-coated dental implants: Anti-inflammatory response and extracellular matrix synthesis. Front. Bioeng. Biotechnol 2023, 11, 1266799. 38116198 10.3389/fbioe.2023.1266799 PMC10728300

[40] Ritz U; Nusselt T; Sewing A; Ziebart T; Kaufmann K; Baranowski A; Rommens PM; Hofmann A The effect of different collagen modifications for titanium and titanium nitrite surfaces on functions of gingival fibroblasts. Clin. Oral Investig 2017, 21, 255–265. 10.1007/s00784-016-1784-5 26969500

[41] Moura CEB; Queiroz Neto MF; Braz J; de Medeiros Aires M; Silva Farias NB; Barboza CAG; Cavalcanti Júnior GB; Rocha HAO; Alves Junior C Effect of plasma-nitrided titanium surfaces on the differentiation of pre-osteoblastic cells. Artif. Organs 2019, 43, 764–772. 30779451 10.1111/aor.13438

[42] Zreiqat H; Valenzuela SM; Nissan BB; Roest R; Knabe C; Radlanski RJ; Renz H; Evans PJ The effect of surface chemistry modification of titanium alloy on signalling pathways in human osteoblasts. Biomaterials 2005, 26, 7579–7586. 16002135 10.1016/j.biomaterials.2005.05.024

[43] Sarvaiya BB; Kumar S; Pathan MSH; Patel S; Gupta V; Haque M The Impact of Implant Surface Modifications on the Osseointegration Process: An Overview. Cureus 2025, 17, e81576. 40177230 10.7759/cureus.81576 PMC11961139

[44] Yi J; Li M; Zhu J; Wang Z; Li X Recent development and applications of electrodeposition biocoatings on medical titanium for bone repair. J. Mater. Chem. B 2024, 12, 9863–9893. 39268681 10.1039/d4tb01081g

[45] Akshaya S; Rowlo PK; Dukle A; Nathanael AJ Antibacterial Coatings for Titanium Implants: Recent Trends and Future Perspectives. Antibiotics 2022, 11, 1719. 36551376 10.3390/antibiotics11121719 PMC9774638

[46] Bonato RS; Fernandes GVO; Calasans-Maia MD; Mello A; Rossi AM; Carreira ACO; Sogayar MC; Granjeiro JM The Influence of rhBMP-7 Associated with Nanometric Hydroxyapatite Coatings Titanium Implant on the Osseointegration: A Pre-Clinical Study. Polymers 2022, 14, 4030. 36235978 10.3390/polym14194030 PMC9570843

[47] Das P; Ganguly S; Marvi PK; Hassan S; Sherazee M; Mahana M; Shirley Tang X; Srinivasan S; Rajabzadeh AR Silicene-Based Quantum Dots Nanocomposite Coated Functional UV Protected Textiles with Antibacterial and Antioxidant Properties: A Versatile Solution for Healthcare and Everyday Protection. Adv. Heal. Mater 2025, 14, e2404911. 10.1002/adhm.202404911 PMC11874647 39757484

[48] He C; Feng P; Hao M; Tang Y; Wu X; Cui W; Ma J; Ke C Nanomaterials in Antibacterial Photodynamic Therapy and Antibacterial Sonodynamic Therapy. Adv. Funct. Mater 2024, 34, 2402588.

[49] Jiang C; Gong G; Xiao S; Zhang S; Chen D; Song S; Dai H; Wu C; Zou Q; Li J; Mechanical and biological properties of 3D-printed porous titanium scaffolds coated with composite growth factors. BMC Oral Health 2025, 25, 808. 40426159 10.1186/s12903-025-06110-2 PMC12107813

